# Hierarchical Activity Recognition Using Smart Watches and RGB-Depth Cameras

**DOI:** 10.3390/s16101713

**Published:** 2016-10-15

**Authors:** Zhen Li, Zhiqiang Wei, Lei Huang, Shugang Zhang, Jie Nie

**Affiliations:** 1 College of Information Science and Engineering, Ocean University of China, Qingdao 266100, China; lizhen@ouc.edu.cn (Z.L.); weizhiqiang@ouc.edu.cn (Z.W.); huangl@ouc.edu.cn (L.H.); zhangshugang@hotmail.com (S.Z.); 2 Department of Computer Science and Technology, Tsinghua University, Beijing 100084, China

**Keywords:** activity recognition, wearable device, RGB-D, hierarchical structure

## Abstract

Human activity recognition is important for healthcare and lifestyle evaluation. In this paper, a novel method for activity recognition by jointly considering motion sensor data recorded by wearable smart watches and image data captured by RGB-Depth (RGB-D) cameras is presented. A normalized cross correlation based mapping method is implemented to establish association between motion sensor data with corresponding image data from the same person in multi-person situations. Further, to improve the performance and accuracy of recognition, a hierarchical structure embedded with an automatic group selection method is proposed. Through this method, if the number of activities to be classified is changed, the structure will be changed correspondingly without interaction. Our comparative experiments against the single data source and single layer methods have shown that our method is more accurate and robust.

## 1. Introduction

Personal lifestyle impacts on our health significantly. For example, some habits like long time sedentary activity or overeating are harmful for our body and lead to many chronic diseases such as diabetes, heart diseases, and hypertension. In order to monitor and evaluate personal health and lifestyle and to discover the relationship between lifestyle and health, an automatic monitoring system is needed to capture personal physical activity data and evaluate personal lifestyle.

In past decades, there have been many methods for activity capture and recognition using different kinds of sensors; the most popular method is using a stationary camera, which is very useful and convenient for activity recognition since there are many closed circuit television systems everywhere. Neil et al. [1] utilized public videos captured from a single RedGreen-Blue (RGB) camera to detect some simple activities such as walking, running, and stopping. Moreover, multiple cameras are introduced in activity recognition (AR) systems. A distributed camera network [2] and a multi-view framework [3] were developed for activity recognition. Further, RGB image data combined with depth data provide more information about activity, so there are many frameworks which were implemented using RGB-D [4,5] data directly or using skeleton [6,7] data extracted from RGB-Depth (RGB-D) data. However, when subjects are out of view of the cameras, image data alone cannot provide any useful information for activity analysis. Aiming at monitoring personal activity 24 h per day, wearable sensors are applied to provide valid data and also assist image data to achieve more accurate recognition of activity.

A wearable device is usually implanted with multiple sensors such as accelerometer, gyroscope, and Global Positioning System (GPS), etc. It is widely used to capture activity data since it records subjects in a free manner no matter where they are located. GPS is also a useful sensor to record the location of the subject, which is important for activity recognition [8]. Also, there are some methods using one or two wearable cameras which are worn on the chest or head for video recording [9]. However, both the GPS and camera are power-consuming sensors which cannot last for a long time within wearable devices. Thus, some existing methods have used one accelerometer or gyroscope to capture movement of the wrist [10,11], hip [12], chest [13], and ankle [14] for activity recognition, while others have placed multiple motion sensors over the whole body [15,16]. The number and position of motion sensors are critical for activity recognition, but it is necessary to balance the recognition accuracy and wearing convenience. Ling at al. [17] compared five positions including thigh, ankle arm, wrist, and hip and claimed that combing both thigh and wrist sensors could get a better results than combing any other sensors. Furthermore, a cell phone is a special type of wearable device which offers a convenient way to monitor lifestyle [18,19]. The cell phone is embedded with many sensors and a transmission module (Wi-Fi/Bluetooth) which can send data wirelessly. However, although wearable devices provide a convenient method for data collection, the data from these sensors is too simple for complicated activity recognition. Moreover, there are some other ambient sensors which are used for indoor activity recognition and healthcare, such as microphones, infrared-ray position sensors, and floor sensors, which are not available in cell phones [20].

**Figure 1:**
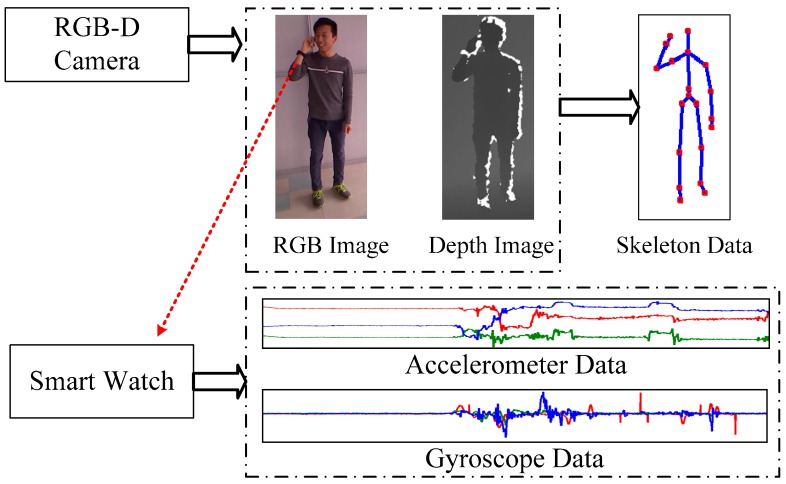
Introduction of the monitoring system.

In order to capture activity data and monitor lifestyle continuously, we have constructed a multi-source daily life monitoring system which consists of wearable smart watches and RGB-D cameras, as shown in Figure 1. The smart watch runs an Android Operating System, which is embedded with an accelerometer, a gyroscope, and a Wi-Fi module for data transmission. The data captured through the watch are shown in Figure 1. Data are sent to a server when network is available. Moreover, one or more fixed RGB-D cameras are utilized for indoor RGB-D image data capture, and the skeleton data [21] is extracted from the depth data. The advantage of choosing a smart watch over traditional sensors is that the watch is a common accessory and so it is comfortable for users to wear. In our system, for indoor cases, RGB-D cameras, which capture rich activity information, combined with motion sensors can easily detect many complicated activities that are undetectable by using motion sensors alone. For outdoor cases, the motion sensor embedded in the smart watch continuously records the wearer’s activities. Thus, combining these two types of devices offers a useful tool for the activity recognition, lifestyle evaluation, and healthcare.

However, there are still some problems that need to be solved using this system. Because there are many data sources in the system, it is common to use a hierarchical framework to combine them together for activity recognition. For example, motion sensor data is utilized at the first layer to separate all activities into a static activity class and a dynamic activity class, and other data sources are introduced at the next layer for further processing. However, the existing hierarchical methods are all hand-designed and cannot be applied to all situations. If the types of activity to be classified are changed, the whole structure has to be changed manually. Moreover, these hierarchical structures are designed using estimates based on experience, and it is not certain whether these structures reflect real situations. Therefore, it is important to design a hierarchical method and an automatic group selection method which could adapt to most general situations.

Moreover, it is possible that there are multiple subjects in the view of one RGB-D camera, and the recognition process will be confused since it cannot judge whether sensor data and skeleton data are from the same subject. In our system, in order to deal with the multi-person situation, a rapid and effective mapping method to bind motion sensor data and image data from the same subject is needed. 

The main contributions of this paper are listed as follows: (1) a novel monitoring system which combines both wearable and fixed devices for activity recognition is proposed. Through this system, we can continuously capture personal daily data; (2) a hierarchical activity recognition structure with an automatic group selection method which combines RGB-D data, accelerometer data, and gyroscope data is proposed. With the help of the new recognition structure, if the types of activity to be classified are changed, then the structure could be changed correspondingly without interaction; (3) a normalized cross correlation (NCC)—based mapping method is proposed to establish association between smart watch data and camera data from the same person in multi-person situations.

There is plenty of previous work related to activity recognition based on motion sensor data, RGB-D data, and skeleton data. Features such as mean, variance, energy, entropy [17], and signal-magnitude area (SMA) [13] were designed for motion sensors. However, with motion sensors, only some simple activities could be detected such as walking, sitting, and running [11,22].

Moreover, a rapid and accurate image feature is a key for activity recognition. Histogram of Oriented Gradient 3-Dimension (HOG3D) [23] and Histogram of Optical Flow 3-Dimension HOF3D [24] have performed well on many datasets. In addition, Kantorov [25] extracted motion information as the feature from compressed video and Fisher vectors were used to encode the feature. Liu et al. [26] proposed a hierarchical partwise bag-of-words feature from both local and global areas. Also, there were many methods using data from RGB-D cameras. Ni et al. [27] proposed a novel feature using 3D spatial and temporal descriptors from both grayscale and depth image channels. The Actionlet Ensemble Model [28] based on depth data and skeleton achieved good performance. However, the fixed cameras restrict the system to track subjects only in limited locations.

Classification methods from generative to discriminative methods are commonly used in activity recognition. The Hidden Markov model is the most common generative approach in activity detection [29,30]. There are many other approaches using a dynamic Bayesian Network [31]. Support Vector Machine (SVM) [32], Boosting [33], Neural Network [34] and Conditional Random Field (CRF) [35] are all discriminative methods utilized in activity recognition. However, one single layer of classification method cannot obtain good results, so some hierarchical methods have been proposed to improve the performance. Khan et al. [13] proposed a two-layer method using Neural Networks based on accelerometer data. Three states, including dynamic, static, and transition, are detected at the first layer, and three classifiers for each state are trained for the second layer. Yin et al. [36] proposed a hierarchical probabilistic latent model which contains four layers using video sequence. However, most of the existing methods use hand-designed hierarchical structures which cannot be applied in varied situations. In addition, if the number and types of activity are changed, the whole structure needs to be adjusted manually. Therefore, our study focuses on providing a general hierarchical method which could generate the recognition strategy automatically from multi-source data.

The remainder of the paper is organized as follows. The method is described in Section 2, and comprehensive experiments are described and comparative results are given in Section 3. Finally, some conclusions are drawn in Section 4.

## 2. Materials and Methods 

### 2.1. Overview 

**Figure 2:**
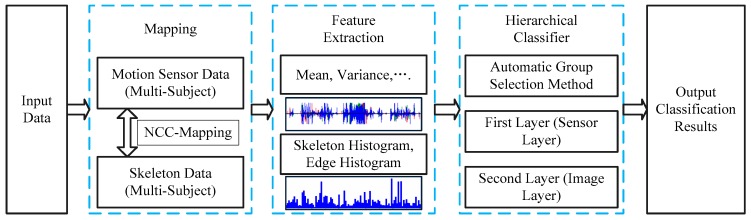
Overview of the proposed method.

The method presented in this paper is illustrated in Figure 2. There are three main steps of the proposed method. In the first step, a Normalized Cross Correlation (NCC) based mapping method is used to bind each skeleton data to its corresponding motion sensor data. In the second step, mean variance, and some other features from motion sensor data, Skeleton Shape Histogram, and Edge Histogram Descriptors from RGB-D data are extracted for activity recognition. At the last step, a hierarchical classifier is constructed, and an automatic group selection method is proposed to build an optimal hierarchical structure to improve the performance of activity recognition. 

### 2.2. Normalized Cross Correlation Mapping Method

**Figure 3:**
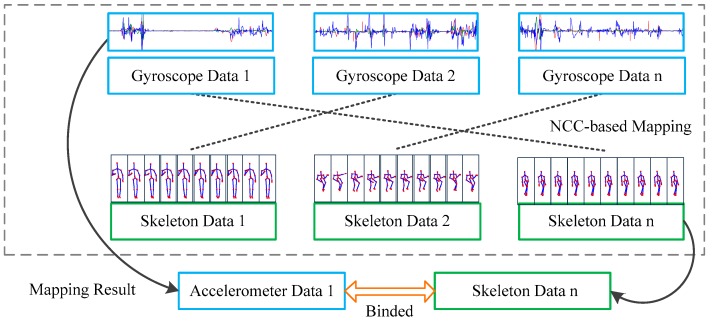
Normalized Cross Correlation (NCC)-based mapping method.

With the help of data from the motion sensor and RGB-D camera, a large amount of activity-related information is acquired. However, when more than one person is captured through a RGB-D camera, their motion sensor data and their corresponding skeleton data should be matched for further processing. Therefore, a mapping method is proposed in this paper to discover the relationship between motion sensor data and skeleton data from a single subject. The process is shown in Figure 3. Both features from gyroscope and skeleton are extracted and an NCC method is introduced for mapping.

First, the feature of total velocity from the gyroscope is extracted, which is calculated using Equation (1):

(1) $$VEL_{gyro}(t)=\sqrt{Gyro_{x}^{2}(t)+Gyro_{y}^{2}(t)+Gyro_{z}^{2}(t)}$$

where $Gyro_{x}(t)$ is the gyroscope data of *x* axis at time *t*. The $VEL_{gyro}(t)$ presents the motion level of the hand of a subject since the smart watch is attached on the wrist.

At the same time, the skeleton data also records the location of the hand in an image sequence. In order to capture the motion information from skeleton data, hand motion velocity is calculated as below:

(2) $$VEL_{image}(t)=\sqrt{v_{x}^{2}(left\_hand,t)+v_{y}^{2}(left\_hand,t)+v_{z}^{2}(left\_hand,t)}$$

(3) $$v_{x}(position,t)=I_{x}(position,t)-I_{x}(position,t-1)$$

where $I_{x}(position,t)$ is the *x* coordinate of the *position* in image at time *t*, and *position* could be any joints such as *head*, *left hand*, *right hand*, *left foot*, *right foot*, and so on. We use *left_hand* in Equation (2) since the smart watch is worn on the left hand. $VEL_{image}(t)$ calculates the velocity of left hand from the image. 

When the velocities of two data sources are available, the NCC method is implemented for two data source mapping and subject identification. The NCC method is defined as:

(4) $$N(t)=\frac{\sum_{t=0}^{T}GT(t)\cdot IT(t)}{\sqrt{\sum_{t=0}^{T}GT^{2}(t)}\sqrt{\sum_{t=0}^{T}IT^{2}(t)}}$$

(5) $$GT(t)=\{\begin{array}{cc} 1 & if\;VEL_{gyro}(t)>T_{gyro}\\ 0 & else \end{array}$$

(6) $$IT(t)=\{\begin{array}{cc} 1 & if\;VEL_{image}(t)>T_{image}\\ 0 & else \end{array}$$

Because of the noise of both data sources, we do not use the original velocity for mapping, instead, two thresholds $T_{gyro}$ and $T_{image}$ are introduced, and GT(t) and IT(t) indicate whether the hand is active or not from different sensors. We find the optimal thresholds $T_{gyro}$ and $T_{image}$ through Equation (7), which provides a simple method to locate these two thresholds.

(7) $$\arg\max_{T_{gyro},T_{image}}(\frac{\sum_{d=1}^{D}GT(d)\cdot IT(d)}{Max(\sum_{d=1}^{D}GT(d),\sum_{d=1}^{D}IT(d))}-\frac{\sum_{d=1}^{D}GT(d)\cdot IT(d)}{D})$$

where *D* is the number of all testing points. $\sum_{d=1}^{D}GT(d)\cdot IT(d)$ calculates the number of matching points which meet two thresholds over all testing points, and $Max(\sum_{d=1}^{D}GT(d),\sum_{d=1}^{D}IT(d))$ calculate the max number of points which meet only one threshold. The results of threshold determination will be illustrated in the experiment section. 

After the threshold determination, for the same skeleton data, different gyroscope data are tested through the proposed NCC-based method and data which obtains the maximum result in Equation (4) are mapped to the skeleton data.

### 2.3. Feature Extraction

#### 2.3.1. Feature of Accelerometer and Gyroscope

In our study, data captured through motion sensors including accelerometer and gyroscope are cut into sub segments which last six seconds. The features of the motion sensor include mean, variance, range, spectral energy, and absolute change (AC), which is defined as follows:

(8) $$AC(x,y,z)=\frac{1}{N}(\sum_{i=1}^{N}\left|x_{i}-x_{i-1}\right|+\sum_{i=1}^{N}\left|y_{i}-y_{i-1}\right|+\sum_{i=1}^{N}\left|z_{i}-z_{i-1}\right|)$$

where $x_{i}$, $y_{i}$ and $z_{i}$ are the *i*th values in *X*, *Y,* and *Z* axis of gyroscope or accelerometer raw data sequence and *N* is the length of the sequence.

#### 2.3.2. Feature of RGB-D Data

RGB-D data are captured through Kinect sensors. The RGB image resolution is 1280 × 960 pixel and the depth image resolution is 640 × 480 pixel. The sampling rate of the RGB images is 12 Hz and that of the depth image is 30 Hz. Skeleton data is extracted from the RGB-D data through Kinect Software Development Kit (SDK). Due to the limitation of the Kinect sensor, the subject cannot be too far away (>5 m) from the sensor. The 3D Shape Histogram feature [37] is utilized for feature extraction. For each skeleton at time *t*, twelve joints including head, left elbow, right elbow, left hand, right hand, left knee, right knee, left foot, right foot, hip center, left hip, and right hip are used for the histogram calculation. Each joint is transferred to a spherical coordinate where the hip center is set as the origin of coordinates. Six seconds skeleton data form a histogram according to their zenith and azimuth angles. The zenith angle is divided into seven equal bins and the azimuth angle is divided into ten equal bins. 

Moreover, Edge Histogram Descriptor (EHD) [38] is extracted around the hand area from RGB image data. The EHD which calculates the edge distribution over hand area is helpful for activity recognition since the activity is highly related to the object which the subject interacts with. Both skeleton histogram and EHD are concentrated as the final feature of RGB-D data.

### 2.4. Hierarchical Recognition Scheme

As long as the motion sensor data is mapped to RGB-D data, all data sources can be obtained for activity recognition. In order to utilize all types of data, a hierarchical method is proposed for activity recognition. Using this structure, when the motion data and image data are all available, the mapping method is used to bind them together, and the activity is recognized through a two-layer hierarchical structure. At the first level, the motion sensor is utilized for classification. All activities can be divided into some groups. At the next layer, image features are introduced. When the subject is out of the scope of the camera, we only use the accelerometer and gyroscope data for activity recognition since the skeleton data is unavailable.

The reason why we chose the hierarchical method is that motion sensor data alone cannot provide enough information because of the limitations and simplicity of its features; specifically, insufficient information is provided for some subtle or complicated activities such as eating or making a call. Therefore, a coarse-fine hierarchical method is used to improve the accuracy rate of the classification. In the hierarchical method, all data are separated into multiple groups at the first layer as the input for the next layer. Finding the optimal groups at the first layer is an important issue. The existing methods all focus on how to design a complicated hierarchical structure, but group selection is only based on experience such as grouping all activities into a static group, dynamic group and transition group. Moreover, once the types of activities are changed, the whole structure cannot be changed correspondingly since the structure is fixed already. So, an automatic group selection method and a hierarchical structure is proposed in this paper.

#### 2.4.1. Automatic Group Selection Method

In this section, we propose a novel automatic group selection method. For the sensor data, we use an SVM classifier for activity recognition. Assuming there are *N* types of activity to be recognized, and the number of groups is *M* = 2 at the beginning, which indicates that all activities will be separated into *M* groups. Then, the performances of all group combinations (GCs) are tested to find the best group combination, and the recognition accuracy of *d*th GC is $T_{d}^{M}$. In the next step, the *M* keeps increasing, and the corresponding $T_{d}^{M}$ is calculated, respectively. When recognition accuracies of all combinations are calculated, the GC with highest $T_{d}^{M}$ is selected as the final GC.

However, the computation complexity of this method is very high since the recognition accuracy rates s of all GCs have to be calculated. We utilized a low time complexity group selection approach, which is shown below:

(9) $$\begin{array}{l} G^{M}=\{C_{1}^{M},C_{2}^{M},...,C_{d}^{M}\}\\ C_{d}^{M}=\{c_{d}^{1},c_{d}^{2},...,c_{d}^{M}\} \end{array}$$

where the set $G^{M}$ contains all possible GCs and $C_{d}^{M}$ is *d*th GC when there are *M* groups, and $c_{d}^{1},c_{d}^{2},...,c_{d}^{M}$ are all groups belonging to a certain $C_{d}^{M}$. For example, when *M* = 2 and *N*=8, it is possible that $c_{d}^{1}$ contains three activities and $c_{d}^{2}$ contains the other five activities. The whole group selection process is shown in Algorithm 1, where $T(c_{d}^{m})$ is the accuracy of group $c_{d}^{m}$, and $Q(C_{d}^{M})$ is the evaluation method for each GC. At first, all activities are divided into two groups, and the recognition accuracy rates s of all GCs are evaluated and the best one is selected as $C_{\max}=\{c_{d}^{1},c_{d}^{2}\}$. Then, the *M* is added to 3 and new GCs are generated based on the previous result $C_{\max}$. For example, if the result of *M*=2 is $c_{d}^{1}=\{a_{1},a_{2},a_{3},a_{4}\}$, $c_{d}^{2}=\{a_{5},a_{6},a_{7},a_{8}\}$, in the next step, we could only split $c_{d}^{1}$ or $c_{d}^{2}$ to generate new GCs. And it is impossible to group $a_{4}$ and $a_{5}$ together since they are not in the same group in the previous result. With the help of this optimal method, the group selection approach does not need to go through all combinations which ease the burden of computation.

**Algorithm 1.** Algorithm of Group Selection**Step 1***N*: Number of types of activities *M* = 2**Step 2**The recognition accuracy rates of all group combinations from $C_{1}^{M}$ to $C_{d}^{M}$ is evaluated. For each $C_{d}^{M}$, it contains *M* classes and a SVM classifier is trained for performance evaluation. $Q(C_{d}^{M})=\frac{\sum_{m}T(c_{d}^{m})}{M}-\frac{1}{M}$ $E_{\max}^{M}=\max(Q(C_{1}^{M}),Q(C_{2}^{M}),...,Q(C_{d}^{M})$ $C_{\max}=\underset{C\in \{C_{1}^{M},C_{2}^{M},...,C_{d}^{M}\}}{\arg\max}(Q(C))$**Step 3**$\begin{array}{cc} If & \left(M=2)\;or\;(E_{\max}^{M}>E_{\max}^{M-1}\;and\;M<N\right)\\ & M=M+1\\ & C^{M}\;\mathrm{is}\;\mathrm{created}\;\mathrm{based}\;\mathrm{on}\;C_{\max}\\ & \mathrm{Repeat}\;\mathrm{step}\;2\\ Else & \mathrm{Stop} \end{array}$

#### 2.4.2. The Recognition Hierarchical Structure 

**Figure 4:**
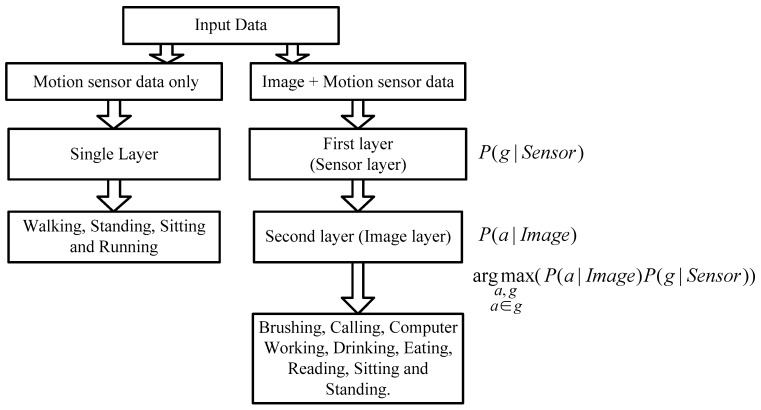
The proposed hierarchical method.

The whole structure of the proposed method is shown in Figure 4. If the subject is out of the view of the camera, then only motion sensor data are available; the single layer SVM classifier is utilized for activity recognition and there are only four kinds of activities that can be detected since motion sensors provide limited information. The four activities are walking, standing, sitting, and running. On the other hand, if both sensor data and image data are available, the proposed hierarchical method is implemented. At the first layer, data from the motion sensor are used for recognition, and features mentioned in the Section 2.3.1 are extracted in this layer including mean, variance, etc. The number of groups and GC depends on the group selection method. The probability of certain group given sensor data P(g|Sensor) is obtained through the SVM classifier with probability output. At the second layer, another SVM classifier involving all activities are trained, and the probability of certain activity *a* given image data P(a|Image) is calculated.

After obtaining two probabilities, the final output is obtained through Equation (10):

(10) $$\mathrm{Activity}=\underset{\begin{array}{l} a,g\\ a\in g \end{array}}{\arg\max(}P(a|\mathrm{Image})P(g|\mathrm{Sensor}))$$

## 3. Results

### 3.1. Dataset 

Ten human subjects including six males and four females, 32 years old on average, participated in the experimental study for data collection. Each subject wore the smart watch and stood in front of a RGB-D camera for data capture, two types of dataset were recorded. One was obtained through subjects acting according to an activity list including the following eight activities which cover a large part of daily life: brushing (BR), calling (CL), computer working (CW), drinking (DK), eating (ET), reading (RD), sitting (ST) and standing (SD). The collected data contained 3-axis gyroscope data, 3-axis accelerometer data, and RGB-D image data. The sampling rate of motion sensors was 50 Hz, the range of the accelerometer was −2 g to +2 g, and the range of gyroscope was −4 rad/s to +4 rad/s. The duration of collecting one activity of one subject was four minutes. The collected data was cut into more than 40 segments and each segment lasted six seconds. More than 400 segments including all data sources of each activity were recorded. To deal with the situation without image data, the other dataset was generated though a simple method. Subjects only wore the smart watch to capture motion data according to an activity list which contained walking, standing, running, and sitting. Similarly, each activity had 400 segments as training and testing samples. The experiment of activity recognition used leave-one-subject-out cross validation protocol.

**Figure 5:**
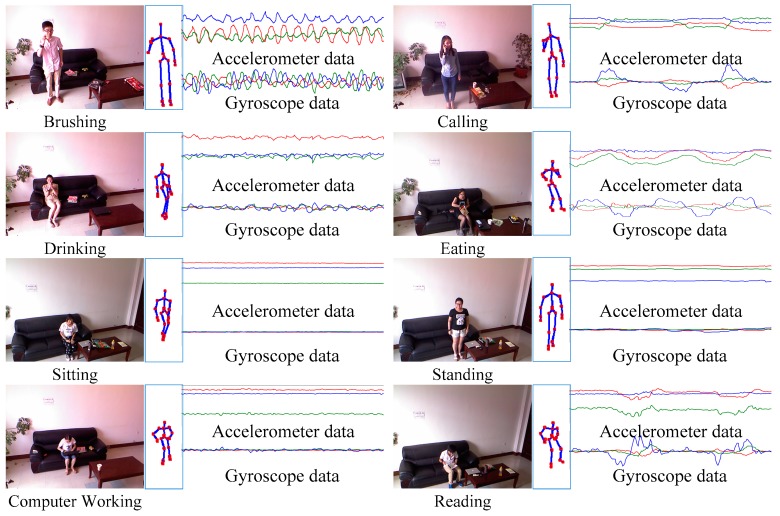
Data collection.

Eight typical activities including all types of data are presented in Figure 5. It can be seen that accelerometer and gyroscope signals and image data have distinctive patterns for activity classification. Moreover, we found that some activities share similar features from motion sensor data such as sitting and computer working.

### 3.2. Thresholds Determination 

**Figure 6:**
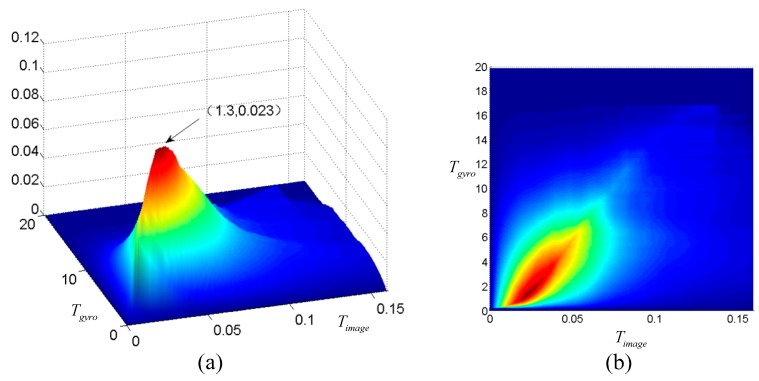
Threshold determination (**a**) 3D view; (**b**) 2D view.

Two thresholds $T_{gyro}$ and $T_{image}$ are used in the NCC-based mapping method to indicate whether the hand is active or not. In this section, we try to find the optimal thresholds. We collected data from both sources for more than five hours for threshold determination. Features of gyroscope and image were extracted through Equations (1) and (2). The thresholds were determined through Equation (7), and the value of Equation (7) with respect to two thresholds is shown in Figure 6. Two thresholds were selected when the surface reached the top, where $T_{gyro}=1.3$ and $T_{image}=0.023$.

### 3.3. Evaluation on Matching Result

The NCC-based mapping method is tested in this section. Eight subjects were involved in the test, and each two subject pairing were asked to act freely in the front of a RGB-D camera. The data was cut into six second sub segments. For each skeleton data, NCC-based mapping was implemented to test all gyroscope data to find the max results in Equation (4) and map it to the skeleton data. It is possible that some NCC results of both sub segments of skeleton data and gyroscope data were close to zero since subjects were inactive at that moment; all these sub segments were removed from the test. 

**Figure 7:**
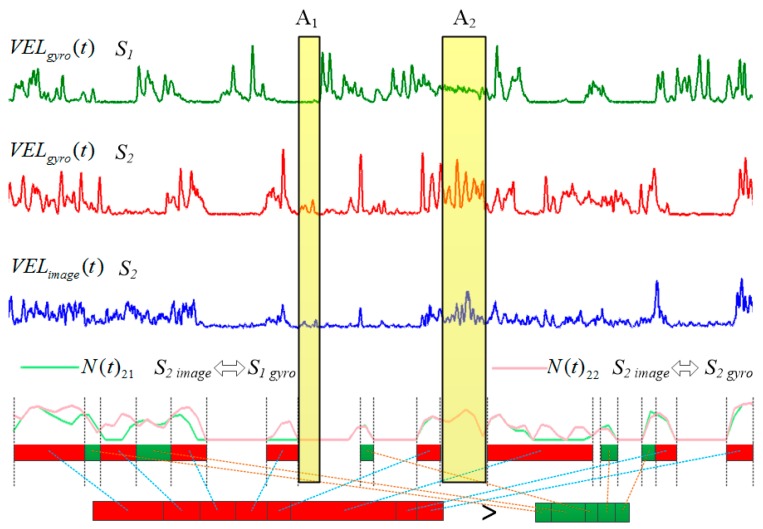
Mapping process from two subjects.

Figure 7 is an example of the mapping process of eight minutes data from two subjects *S_1_* and *S_2_*, the first curve is the velocity of the gyroscope data from *S_1_* and the second curve is that from *S_2_*. The third curve is the velocity of the skeleton data from *S_2_*. All velocity data were processed by a media filter to remove noise. The second and third curves are very similar since they come from the same subject. Two series of N(t) values are calculated during a relative long time period respectively, the first one is between $S_{2\;image}$ (image data of *S_2_*) and $S_{1\;gyro}$ (gyroscope data of *S_1_*), denote as $N{\left(t\right)}_{21}$, illustrated as light green curve and the second is between $S_{2\;image}$ and (gyroscope data of *S_2_*) $S_{2\;gyro}$, denote as $N{\left(t\right)}_{22}$, illustrated as light red curve. Then each corresponding values in two series are compared and number of higher values is recorded. The length of green bar is the number of higher values of $N{\left(t\right)}_{21}$. Similarly, the length of red bar is the number of higher values of $N{\left(t\right)}_{22}$. $S_{2\;image}$ and $S_{2\;gyro}$ which come from the same subject constructed the serie with more number of higher values (the red bar) are selected as a pair since it indicates a higher match degree. 

Normally, we tracked more than 15 min data for mapping. But it was possible that both subjects preformed similar activities or kept still at the same time, such as A_1_ and A_2_ intervals in Figure 7. Both subjects kept still in the A_1_ interval and performed similar active activities in the A_2_ interval. The N(t) values from the two subjects are very close or the same in these situations, and these intervals are denoted as invalid intervals for mapping. However, in our method we tracked a long time period data sequence and it is impossible that all subjects always perform similarly, so these invalid intervals do not affect the mapping results.

**Figure 8:**
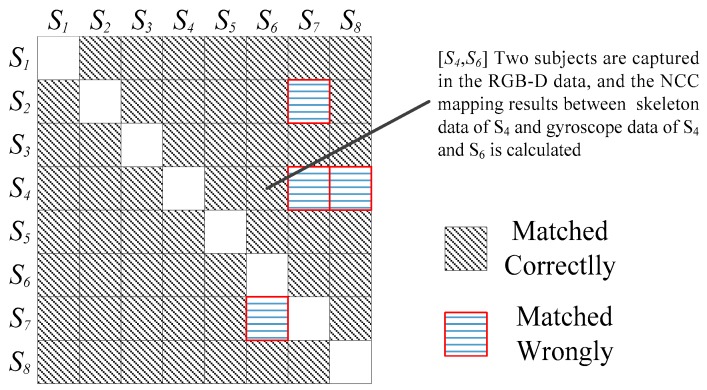
Results of NCC-based mapping.

$S_{1}-S_{8}$ are denoted as eight subjects, each cell $\left[S_{x},S_{y}\right]$ is the NCC-based mapping result between skeleton data of one subject $S_{x}$ and the gyroscope data of two subjects ($S_{x}$ and $S_{y}$). From the figure, there are 52 samples matched correctly while four samples matched incorrectly. The accuracy of the mapping method is 92.86% (52/56). For the four missed samples, most of them are matched incorrectly since the skeleton extraction method was inaccurate and the position of skeleton hand data shifted to other places so it was difficult to find the active segment. Figure 8 shows the mapping results of eight subjects.

### 3.4. Results of Group Selection Method

**Figure 9:**
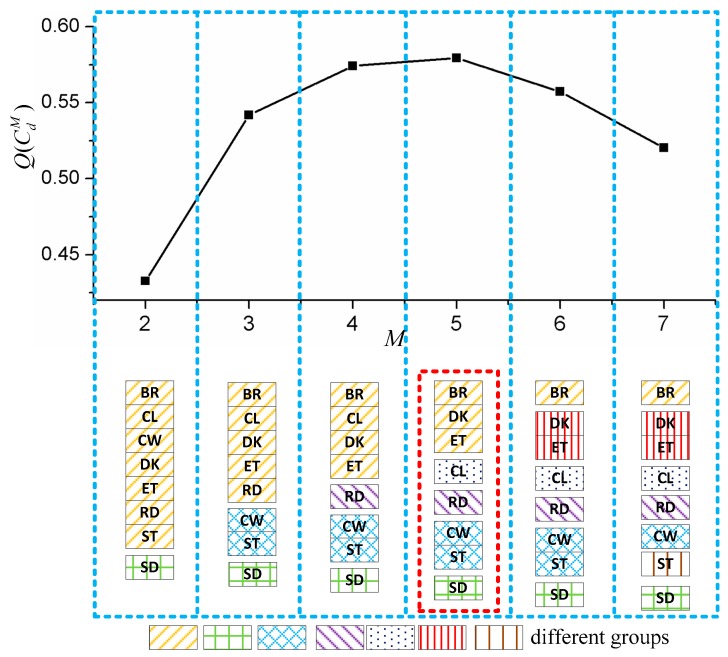
Results of group selection method.

The group selection method is proposed in the Section 2.4.1, and results are shown in Figure 9. The top part of the figure is the curve of the value of $Q(C_{d}^{M})$ with respect to *M* based on the data of motion sensors. When *M* equals 5, the curve reaches the highest point among other GCs, which indicates that five groups, which are shown in the red rectangle, are the optimal GC. When *M* equals 2, only standing is separated from other activities. It is possible that one axis of the accelerometer of the watch is parallel to the direction of gravity when subjects are standing, so the mean value of the accelerometer during the standing activity is different from that during other activities. When *M* equals 3, both computer working and sitting are selected since subjects remain still in these two activities. In the next round, the reading is picked out from previous set since flipping pages shows representative features from motion data. Finally, when M=5, calling is separated from other activities and the value of $Q(C_{d}^{M})$ reaches the top because subjects always kept their hand still and were standing when they made a call. 

### 3.5. Performance on the Proposed Method

The summed confusion matrix from the leave-one-subject-out cross validation is shown in Table 1.The *F1* measurement [39] is used for evaluation, which is implemented as below:

$$\begin{array}{c} \mathrm{Precision}=TP/(TP+FP)\\ \mathrm{Recall}=TP/(TP+FN)\\ F1=2\times \mathrm{Precision}\times \mathrm{Recall}/(\mathrm{Precision}+\mathrm{Recall}) \end{array}$$

where *TP* is the true positive, *FP* is the false positive and *FN* is the false negative. This definition indicates that *F1* is the harmonic mean of recall and precision.

The average *F1* value is 0.839. The *F1* value of standing is highest since it is easily detected by the motion sensor. The mean feature and variance feature are representative for detection. Moreover, the performances of computer working and sitting are good. Although these two activities are grouped together at the first layer because of the similar features of motion sensor data, the image feature is helpful to distinguish them through the area around the hands. The *F1* value of brushing, calling, and reading are over 0.8 since both motion sensor and image data play important roles in detecting these activities. However, results of drinking and eating are not very good because they are grouped into the same group. The differences between these two kinds of activity from skeleton data are too slight.

**Figure 10:**
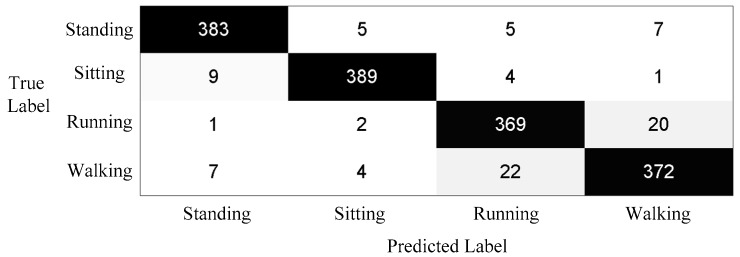
Summed confusion matrix results of motion sensor.

Results of activity recognition when subjects are out of the view of the cameras are shown in Figure 10. Similarly, the experiment was implemented using leave-one-subject-out cross validation. Ten subjects are involved in this part, and each subject acts four kinds of activity according to an activity list. Similarly, each activity was cut by six seconds. Because motion sensor data provides limited information and cannot distinguish complicated activities, only four kinds of activities were detected including standing, sitting, running, and walking. The average *F1* value is 0.946 since the motion sensor is robust to detect these simple activities. 

### 3.6. Comparison between the Proposed Method and Single Layer Method 

**Figure 11:**
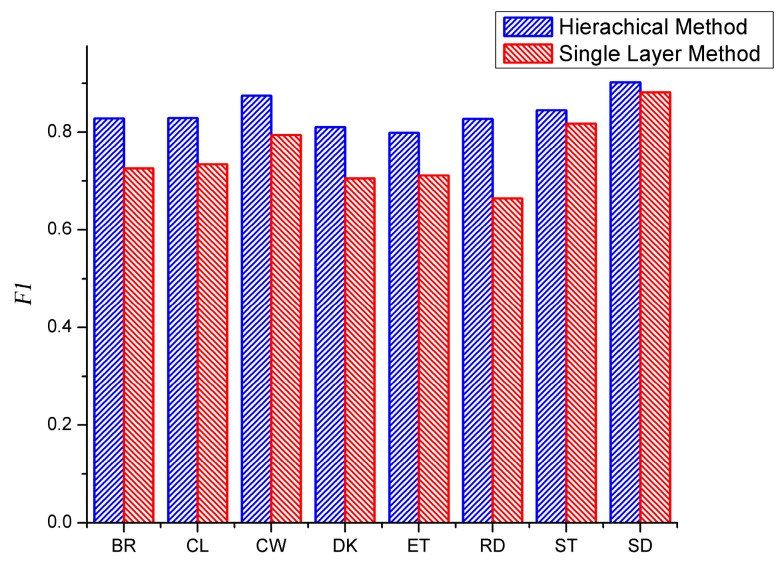
Comparison between the hierarchical method and single layer method.

A hierarchical structure is proposed in this paper. Results of single layer method without the group selection method are presented to compare with the proposed method as shown in the Figure 11. In the single layer method, motion feature and image feature are concentrated together and an SVM classifier is used for recognition. It can be observed from Figure 11 that, in all cases, the proposed method performs better than the single layer method. The *F1* value of reading of the hierarchical method is much higher than that of the single layer method since the reading needs to be classified among all eight activities through the single layer method and a large part of reading activities were misclassified as computer working or sitting, while these two activities belong to another group using the proposed method. Similarly, the result of computer working through the proposed method performs better than the other method. The results of standing and sitting activities of the two methods are close since the features from both sources are discriminative. 

### 3.7. Comparison between the Data Fusion and Single Source Data

**Figure 12:**
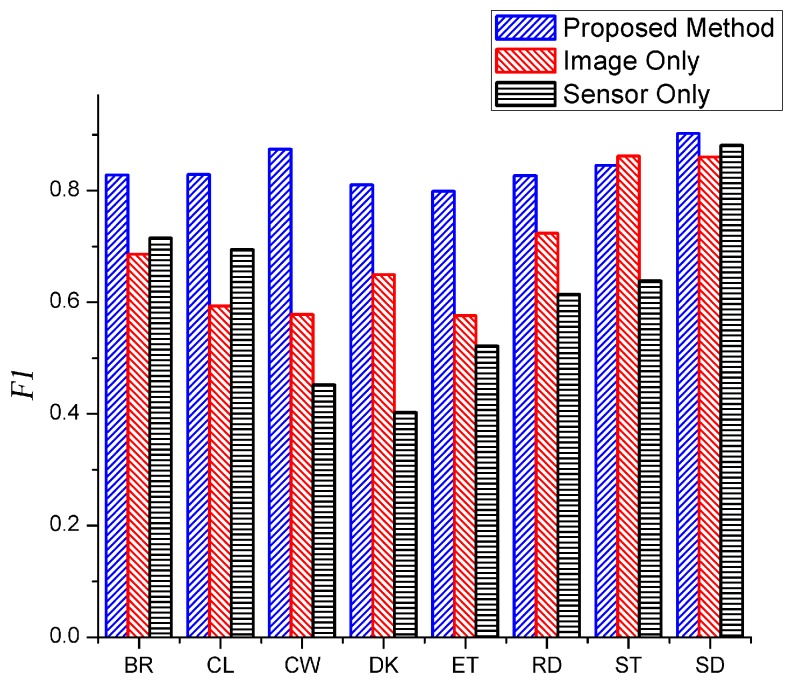
Comparison between the data fusion and single source data.

In order to evaluate whether combining two data sources improves the performance and accuracy of activity recognition, we split the dataset into two datasets. One method only used the motion sensor data and the other method only used image data; a linear SVM was utilized for training and testing. The results are shown in Figure 12. It is concluded that combining both data sources performs better than using each single data source. Standing gets high *F1* value among all three datasets. The *F1* average value of image is higher than that of sensor, since image provides more information concerning activity. For example, the results of computer working and drinking based on the image dataset are higher than that based on the sensor dataset, it is possible that objects which the user interacts with are utilized in the image data. On the other hand, sensor data performs better in detecting calling activity while a lot of calling activities were classified as brushing through image data since image and skeleton data cannot capture tiny actions when the subject is far away from the camera. 

## 4. Conclusions

In this paper, a hierarchical activity recognition method is proposed. A capturing system was constructed for data capture. In this system, a smart watch attached to the subject was used to capture motion senor data for a whole day, and the RGB-D cameras were used to capture image data and skeleton data indoors. Combining both types of data provides rich information and a novel way for data collection and activity recognition.

In this system, it is possible that there were two or more subjects in the view of the camera. In order to map motion sensor data to its corresponding skeleton data, an NCC-based mapping method was implemented for data binding. If the subject was out of the view of the camera, we could not find his corresponding skeleton data. In this case, only four types of activity were recognized including standing, sitting, walking, and running based on motion sensor data only. Otherwise, the hierarchical recognition method was implemented.

In the hierarchical method, a two-layer activity recognition structure was built. The first layer only used motion sensor data, and in order to utilize all data sources effectively, a group selection method was proposed. Instead of designing the structure manually according to experience, the group selection method finds the optimal group combination automatically and builds the hierarchical recognition structure. With this method, if the activities to be classified are changed, the structure will change its group combination and structure automatically without interaction. Our experimental results demonstrate that the proposed algorithm performs better than other methods which use only single layer or single data sources.

## Figures and Tables

**Table 1:** Summed confusion matrix from the leave-one-subject-out cross validation.

		Predicted Label							
		BR	CL	CW	DK	ET	RD	ST	SD
True Label	BR	301	66	0	10	0	0	1	22
	CL	16	348	0	0	2	3	0	31
	CW	0	9	355	0	12	8	16	0
	DK	1	1	18	321	37	12	7	3
	ET	0	1	15	29	327	17	11	0
	RD	0	1	10	20	30	318	21	0
	ST	2	5	14	13	11	11	335	9
	SD	7	9	0	0	0	0	2	382
	*F1*	0.828	0.829	0.874	0.810	0.799	0.827	0.845	0.902

## References

[1] Robertson N., Reid I. (2006). A general method for human activity recognition in video. Comput. Vis. Image Underst..

[2] Bi S., Ahmed K.T., Cristian S., Chong D., Farrell J.A. (2010). Roy-Chowdhury Amit K Tracking and Activity Recognition through Consensus in Distributed Camera Networks. IEEE Trans. Image Process..

[3] Mosabbeb E.A., Raahemifar K., Fathy M. (2013). Multi-view human activity recognition in distributed camera sensor networks. Sensors.

[4] Zhang C., Tian Y. (2012). Rgb-d camera-based daily living activity recognition. J. Comput. Vis. Image Process..

[5] Ni B., Wang G., Moulin P. (2013). GBD-HuDaAct: A color-depth video database for human daily activity recognition. Adv. Comput. Vis. Pattern Recognit..

[6] Fotiadou E., Nikolaidis N. A correspondence based method for activity recognition in human skeleton motion sequences. Proceedings of the 2014 IEEE International Conference on Image Processing (ICIP).

[7] Zhu G., Zhang L., Shen P., Song J. (2016). An Online Continuous Human Action Recognition Algorithm Based on the Kinect Sensor. Sensors.

[8] Huang W., Li M., Hu W., Song G. Cost sensitive GPS-based activity recognition. Proceedings of the International Conference on Fuzzy Systems and Knowledge Discovery.

[9] Sun M., Burke L.E., Baranowski T., Fernstrom J.D., Zhang H., Chen H.C., Bai Y., Li Y., Li C., Yue Y. (2015). An exploratory study on a chest-worn computer for evaluation of diet, physical activity and lifestyle. J. Healthc. Eng..

[10] Chernbumroong S., Atkins A.S., Yu H. Activity classification using a single wrist-worn accelerometer. Proceedings of the 2011 5th International Conference on Software, Knowledge Information, Industrial Management and Applications (SKIMA).

[11] Garcia-Ceja E., Brena R.F., Carrasco-Jimenez J.C., Garrido L. (2014). Long-Term Activity Recognition from Wristwatch Accelerometer Data. Sensors.

[12] Rosenberger M.E., Haskell W.L., Albinali F., Mota S., Nawyn J., Intille S. (2013). Estimating activity and sedentary behavior from an accelerometer on the hip or wrist. Med. Sci. Sports Exerc..

[13] Khan A.M., Lee Y.-K., Lee S.Y., Kim T.-S. (2010). A triaxial accelerometer-based physical-activity recognition via augmented-signal features and a hierarchical recognizer. IEEE Trans. Inf. Technol. Biomed..

[14] Mannini A., Intille S.S., Rosenberger M., Sabatini A.M., Haskell W. (2013). Activity recognition using a single accelerometer placed at the wrist or ankle. Med. Sci. Sports Exerc..

[15] Gao L., Bourke A.K., Nelson J. A comparison of classifiers for activity recognition using multiple accelerometer-based sensors. Proceedings of the IEEE 11th International Conference on Cybernetic Intelligent Systems.

[16] Zhu C., Sheng W. Human daily activity recognition in robot-assisted living using multi-sensor fusion. Proceedings of the IEEE International Conference on Robotics and Automation.

[17] Bao L., Intille S.S. Activity recognition from user-annotated acceleration data. Proceedings of the Second International Conference on Pervasive Computing.

[18] Morillo L.M.S., Gonzalez-Abril L., Ramirez J.A.O., de la Concepcion M.A.A. (2015). Low Energy Physical Activity Recognition System on Smartphones. Sensors.

[19] Inoue S., Hattori Y. Toward high-level activity recognition from accelerometers on mobile phones. Proceedings of the 4th International Conference on Cyber, Physical and Social Computing.

[20] Silva L.C.D., Morikawa C., Petra I.M. (2012). State of the art of smart homes. Eng. Appl. Artif. Intell..

[21] Shotton J., Fitzgibbon A., Cook M., Sharp T., Finocchio M., Moore R., Kipman A., Blake A. Real-time human pose recognition in parts from single depth images. Proceedings of the 2011 IEEE Conference on Computer Vision and Pattern Recognition (CVPR).

[22] Liang Y., Zhou X., Yu Z., Guo B. (2014). Energy-efficient motion related activity recognition on mobile devices for pervasive healthcare. Mob. Networks Appl..

[23] Klaser A., Marszalek M., Schmid C. A spatio-temporal descriptor based on 3d-gradients. Proceedings of the British MachineVision Conference.

[24] Holte M.B., Chakraborty B., Gonzalez J., Moeslund T.B. (2012). A Local 3-D Motion Descriptor for Multi-View Human Action Recognition from 4-D Spatio-Temporal Interest Points. IEEE J. Sel. Top. Signal Process..

[25] Kantorov V., Laptev I. Efficient Feature Extraction, Encoding, and Classification for Action Recognition. Proceedings of the 2014 IEEE Conference on Computer Vision and Pattern Recognition (CVPR).

[26] Liu A.-A., Su Y.-T., Jia P.-P., Gao Z., Hao T., Yang Z.-X. (2015). Multipe/Single-View Human Action Recognition via Part-Induced Multitask Structural Learning. IEEE Trans. Cybern..

[27] Ni B., Yong P., Moulin P., Yan S. (2013). Multilevel depth and image fusion for human activity detection. IEEE Trans. Cybern..

[28] Wang J., Liu Z., Wu Y., Yuan J. Mining actionlet ensemble for action recognition with depth cameras. Proceedings of the 2012 IEEE Conference on Computer Vision and Pattern Recognition (CVPR).

[29] Mannini A., Sabatini A.M. (2011). Accelerometry-based classification of human activities using Markov modeling. Comput. Intell. Neurosci..

[30] Boutell M., Brown C. (2006). Pictures are not taken in a vacuum—An overview of exploiting context for semantic scene content understanding. IEEE Signal Process. Mag..

[31] Oliver N., Garg A., Horvitz E. (2004). Layered representations for learning and inferring office activity from multiple sensory channels. Comput. Vis. Image Underst..

[32] Yin J., Yang Q., Pan J.J. (2008). Sensor-based abnormal human-activity detection. IEEE Trans. Knowl. Data Eng..

[33] Shimosaka M., Mori T., Sato T. Robust indoor activity recognition via boosting. Proceedings of the 2008 19th International Conference on Pattern Recognition.

[34] Zeng M., Nguyen L.T., Yu B., Mengshoel O.J., Zhu J., Wu P., Zhang J. Convolutional Neural Networks for Human Activity Recognition Using Mobile Sensors. Proceedings of the 2014 6th International Conference on Mobile Computing, Applications and Services (MobiCASE).

[35] Cao L., Luo J., Kautz H., Huang T.S. (2009). Image Annotation Within the Context of Personal Photo Collections Using Hierarchical Event and Scene Models. IEEE Trans. Multimed..

[36] Yin J., Meng Y. Human activity recognition in video using a hierarchical probabilistic latent model. Proceedings of the 2010 IEEE Computer Society Conference on Computer Vision and Pattern Recognition Workshops.

[37] Xia L., Chen C.C., Aggarwal J.K. View invariant human action recognition using histograms of 3D joints. Proceedings of the 2012 IEEE Computer Society Conference on Computer Vision and Pattern Recognition Workshops.

[38] Yang M.Y.M., Serrano J.C., Grecos C. (2009). MPEG-7 Descriptors Based Shot Detection and Adaptive Initial Quantization Parameter Estimation for the H.264/AVC. IEEE Trans. Broadcast..

[39] Li Z., Wei Z., Yue Y., Wang H., Jia W., Burke L.E., Baranowski T., Sun M. (2015). An Adaptive Hidden Markov Model for Activity Recognition Based on a Wearable Multi-Sensor Device. J. Med. Syst..

